# 磁性氟化共价三嗪骨架材料用于高效萃取全氟化合物

**DOI:** 10.3724/SP.J.1123.2025.07004

**Published:** 2026-04-08

**Authors:** Wenmin ZHANG, Guancheng LIU, Min FANG, Junling GUO, Lan ZHANG

**Affiliations:** 1.闽江师范高等专科学校，福建 福州 350108; 1. Minjiang Teachers College，Fuzhou 350108，China; 2.福州大学，福建 福州 350116; 2. Fuzhou University，Fuzhou 350116，China

**Keywords:** 共价三嗪骨架, 全氟化合物, 磁性固相萃取, 高效液相色谱-串联质谱, covalent triazine framework （CTF）, perfluorinated compounds （PFCs）, magnetic solid-phase extraction （MSPE）, high performance liquid chromatography-tandem mass spectrometry （HPLC-MS/MS）

## Abstract

全氟化合物（PFCs）是一种新型的持久性有机污染物，消费者摄入PFCs污染的软饮料后，会引起严重的系统性疾病。然而，软饮料中PFCs的含量极低且基质复杂，直接利用高效液相色谱-串联质谱（HPLC-MS/MS）进行含量测定比较困难，因此在仪器分析之前需要对样品进行必要的前处理。本研究通过一锅法成功制备了一种磁性氟化共价三嗪骨架材料（Fe_2_O_3_/CTF-F），并将其作为磁性固相萃取（MSPE）技术的吸附剂，用于7种PFCs的高效萃取。同时，系列表征结果显示Fe_2_O_3_/CTF-F拥有高比表面积（1 452.3 m^2^/g）、高孔隙度（0.82 cm^3^/g）、高含氟量（17.50%）和强磁响应性（7.1 emu/g），可以提供大量的可接触吸附位点、高亲和的氟-氟作用力以及快速的磁性分离能力，对PFCs表现出优异的萃取能力。随后，通过单因素实验，优化了吸附剂的用量、提取时间、洗脱溶剂和洗脱时间等MSPE过程中的关键条件，并将其与HPLC-MS/MS技术相结合，建立了一种用于软饮料中PFCs分析的新方法。所建立的分析方法具有线性范围宽（0.008~250.0 pg/mL）、线性相关性高（*R*≥0.999 2）、检出限低（0.002~0.005 pg/mL）、重复性良好（RSD≤8.2%， *n*=5）等优点。最后，通过所建立的分析方法对5种软饮料样品进行了分析，并且在所有样品中均检测出了超痕量的PFCs，含量范围为3.5~54.6 pg/mL。实验结果表明，Fe_2_O_3_/CTF-F是一种极具潜力的吸附剂，可用于PFCs的高效萃取，同时所建立的分析方法也适用于软饮料中PFCs的高灵敏检测。

## Abstract (en)

Perfluorinated compounds （PFCs） are a new type of persistent organic pollutants. When consumers consume soft drinks contaminated with PFCs， it can cause severe systemic diseases. Increasing concern regarding the presence of PFCs in soft drinks has resulted in the need for reliable analytical methods for the monitoring of PFCs. However， the content of PFCs in soft drinks is extremely low and the matrix is complex， making it difficult to directly determine their content using high performance liquid chromatography-tandem mass spectrometry （HPLC-MS/MS）. Therefore， the necessary sample pretreatment is required before instrumental analysis. Magnetic solid-phase extraction （MSPE） is a simple， rapid， and efficient solid-liquid separation technique， and its extraction efficiency depends on the characteristics of the magnetic adsorbent. Covalent triazine frameworks （CTFs） are porous organic polymers connected by triazine bonds. They have the characteristics of large specific surface area， rich pore structure， adjustable functionality， good chemical stability and thermal stability， making them widely used in sample pretreatment. In this study， a magnetic fluorinated covalent triazine framework material （Fe_2_O_3_/CTF-F） was prepared by a one-pot method and characterized in detail. The X-ray photoelectron spectra （XPS）， Fourier-transform infrared （FT-IR） spectrum， and X-ray diffraction （XRD） pattern demonstrated the successful synthesis of Fe_2_O_3_/CTF-F with a high fluorine content （17.50%）， which was formed through tetrafluoroterephthalonitrile polymerization and hydrated ferric chloride decomposition. The highly fluorinated material can provide a specific and strong affinity for PFCs through fluorous-fluorous （F-F） interactions. The experimental results of nitrogen adsorption-desorption and magnetic properties showed that the Fe_2_O_3_/CTF-F has a high specific surface area （1 452.3 m^2^/g） and porosity （0.82 cm^3^/g）， as well as a strong magnetic responsivity （7.1 emu/g）. It indicates that the Fe_2_O_3_/CTF-F possesses a large number of accessible adsorption sites and a rapid magnetic separation capability， providing a guarantee for efficient extraction of PFCs. Subsequently， the Fe_2_O_3_/CTF-F was used as an adsorbent for MSPE technology for the efficient extraction of seven PFCs. Because of its many accessible adsorption sites and strong fluorine-fluorine interactions， the Fe_2_O_3_/CTF-F showed excellent extraction ability for PFCs. Finally， by combining the MSPE method with HPLC-MS/MS technology， a new analytical method was established for the analysis of PFCs in soft drinks. To achieve the best performance， single-factor experiments were conducted to optimize the dosage of the adsorbent， the extraction time， the elution solvent and elution time in the MSPE process. Under the optimal conditions， the established analytical method has the advantages of wide linear ranges （0.008–250.0 pg/mL）， high linear correlations （*R*≥0.999 2）， low LODs （0.002–0.005 pg/mL）， and good repeatability （RSDs≤8.2%， *n*=5）. The established analytical method was then used to analyze five kinds of soft drink samples， and ultra-trace amounts of PFCs were detected in all samples， with contents ranging from 3.5 to 54.6 pg/mL. Among them， the highest content of perfluorooctanoic acid （PFOA） measured was 54.6 pg/mL， and its content did not exceed the limit stipulated in the national standard GB 5749-2022 （80 pg/mL）. Besides， the established analytical method was also compared comprehensively with other reported methods. The established method only requires a small amount of adsorbent （5.0 mg）， and can achieve a low detection limit （0.002 pg/mL） after a short pretreatment time （20 min）. The comparison results indicate that the established method has the advantages of being rapid， sensitive， and accurate， and can be used for the monitoring of PFCs in soft drinks. Meanwhile， the preparation method of the Fe_2_O_3_/CTF-F in this study is very simple and convenient， which is conducive to the transformation and application of this method. In conclusion， the Fe_2_O_3_/CTF-F is a highly potential adsorbent for the efficient extraction of PFCs， and the established analytical method is also suitable for the high-sensitivity detection of PFCs in soft drinks.

全氟化合物（perfluorinated compounds， PFCs）是一类碳链上的氢元素完全被氟元素取代的化合物。它具有优异的稳定性和表面活性，已被广泛用于纺织、造纸、食品包装、消防等领域^［1-3］^。但是，PFCs由于其持久稳定性和生物蓄积性，已被列入持久性有机污染物（persistent organic pollutants， POPs）目录中^［4］^。消费者摄入PFCs污染的食品后，会引起严重的免疫、神经、生殖等系统疾病。在众多消费品中，软饮料广受大众欢迎，在全球范围内消费量巨大，仅在2019年中国的人均软饮料年消费量就高达14.9 L^［5］^。因此，对软饮料中的PFCs进行检测具有重要意义。然而，由于PFCs在软饮料中的浓度极低以及复杂的基质效应，在使用高效液相色谱-串联质谱仪（HPLC-MS/MS）进行分析之前，需要对样品进行有效的预处理^［6-8］^。

磁性固相萃取（magnetic solid-phase extraction， MSPE）是一种简便、快速、高效的固液分离技术，可以在外加磁场的作用下实现目标物从样品基质中快速分离^［9，10］^。由于其特殊的磁性分离机制，MSPE在样品预处理应用中受到了广泛关注^［11-17］^。在该技术中，磁性吸附剂的选择性对萃取效率有着至关重要的作用。就PFCs而言，利用氟-氟（F-F）相互作用是提高其萃取效率的有效手段之一。F-F相互作用是发生在氟化分子之间的非共价键力。研究表明，氟功能化的吸附剂对PFCs具有优异的选择性。例如，Ye等^［18］^制备了氟功能化的磁性介孔微球，用于选择性萃取环境水中的PFCs；Zhang等^［4］^制备了磁性氟化共价有机骨架材料，用于牛奶中PFCs的选择性富集。虽然这些方法都取得了良好的萃取效果，但磁性氟化吸附剂的制备过程过于复杂且耗时，不利于其作为 MSPE 吸附剂的推广和应用^［19，20］^。因此，开发易于制备的磁性氟化吸附剂对选择性萃取PFCs具有重要意义。

共价三嗪骨架（covalent triazine frameworks， CTFs）材料是一种由三嗪键连接的多孔有机聚合物。CTFs具有比表面积大、孔隙结构丰富、化学稳定性和热稳定性良好、功能可调等特点，在样品预处理领域广泛应用^［21-24］^。与共价有机骨架材料等相比，CTFs具有更好的耐高温性能（最高可达500 ℃），可以通过微波辅助离子加热的方式，使含有氰基的有机单体发生缩聚反应，从而快速制备CTFs^［21，23］^。因此，利用CTFs作为氟元素的载体，通过F-F相互作用来萃取PFCs是一个很好的选择。此外，由于氯化铁在高温下可以分解生成氧化铁，所以将其作为原材料之一，还能赋予吸附剂所需的磁性能^［25］^。

综上所述，本研究尝试采用微波辅助一锅法制备磁性氟化共价三嗪骨架（Fe_2_O_3_/CTF-F）材料，并将其作为MSPE吸附剂，用于软饮料中7种PFCs的高效萃取。同时，本实验还考察了MSPE萃取过程的主要影响因素。最后，将MPSE与HPLC-MS/MS相结合，建立了一种用于软饮料中7种PFCs分析的新方法。

## 1 实验部分

### 1.1 仪器、试剂与材料

Accela HPLC-TSQ Quantum Access Max高效液相色谱-三重四极杆质谱联用仪、Nicolet iS50傅里叶红外光谱仪（FT-IR）、K-Alpha+X-射线光电子能谱仪（XPS）（美国Thermo Fisher公司）；HT7700透射电子显微镜（TEM，日本Hitachi公司）；D8 Advance X-射线衍射分析仪（XRD，德国Bruker公司）；ASAP 2020 氮气吸-脱附测定仪（美国Micromeritics公司）；PPMS-9振动样品磁强计（VSM，英国Quantum Design公司）；MAS-Ⅱ Plus微波合成仪（上海新仪微波化学科技有限公司）。

无水氯化锌（≥98.0%）、四氟对苯二腈（≥99.0%）、六水合氯化铁（≥99.0%）、盐酸（37.0%）、乙醇（≥99.5%）、甲酸铵（≥98.0%）、甲酸（FA，≥99.5%）和甲醇（≥99.5%）均购自上海国药集团化学试剂有限公司；全氟丁酸（PFBA，≥98.0%）、全氟庚酸（PFHpA，≥98.0%）、全氟己酸（PFHxA，≥98.0%）、全氟辛酸（PFOA，≥98.0%）、全氟戊酸（PFPeA，≥98.0%）、全氟癸酸（PFDA，≥98.0%）和全氟壬酸（PFNA，≥96.5%）均购自美国Sigma-Aldrich公司。实验用水为超纯水（18.2 MΩ·cm， 美国Milli-Q公司）。

### 1.2 实验方法

#### 1.2.1 标准溶液的配制

7种PFCs标准储备液（100.0 μg/mL）分别用甲醇配制，在‒20 ℃下保存。PFCs混合标准溶液储备液（10.0 μg/mL）由上述标准溶液储备液用甲醇稀释混匀配制，在4 ℃下保存。系列混合标准溶液使用超纯水逐级稀释配制，现配现用。

#### 1.2.2 Fe_2_O_3_/CTF-F材料的合成

称取0.3 g六水合氯化铁于瓷坩埚中，加入少许乙醇溶解。随后依次加入3.0 g四氟对苯二腈和10.6 g无水氯化锌，并搅拌均匀。将所获得的混合物转移放入微波合成仪（功率300 W）中反应30 min。待产物冷却至室温后，先用稀盐酸洗涤除去氯化锌，然后用超纯水和乙醇交替清洗3次，最后在100 ℃下真空干燥24 h，获得Fe_2_O_3_/CTF-F材料。

#### 1.2.3 样品前处理

软饮料样品购自当地超市，包括矿泉水、果汁、汽水、能量饮料和茶饮料。所有样品先用0.45 μm滤膜过滤，再用超纯水稀释10倍之后使用。

#### 1.2.4 MSPE

称取5.0 mg Fe_2_O_3_/CTF-F加入20 mL稀释后的实际样品中，以300 r/min的速率振荡10 min。随后，在外加磁场的作用下使Fe_2_O_3_/CTF-F与基质分离，并去除上层清液。接着加入1.0 mL 3%甲酸甲醇溶液涡旋10 min，将PFCs从Fe_2_O_3_/CTF-F上洗脱下来。最后，洗脱液经磁性分离收集后，进行HPLC-MS/MS分析。

#### 1.2.5 HPLC-MS/MS分析条件

Thermo Scientific Accucore保护柱（50 mm×2.1 mm， 2.6 μm）；Thermo Fisher Hypersil GOLD 分析柱（150 mm×2.1 mm， 5 μm）；流速：200 μL/min；柱温：室温；进样量：10 μL。流动相A为5 mmol/L甲酸铵水溶液，流动相B为纯甲醇。梯度洗脱：0~8 min，60%B~90%B；8~9 min，90%B~60%B；9~12 min，60%B。

采用电喷雾电离源（ESI）负离子模式；喷雾电压：‒2 200 V；汽化温度：270 ℃；毛细管温度：300 ℃；以氮气（≥99.999%）为辅助气和鞘气，流速分别为35 arb和10 arb；以氩气（≥99.999%）为碰撞气；采用选择反应监测模式（SRM）用于定量分析。7种PFCs的离子对、碰撞电压和透镜电压由仪器自动调谐获得，具体参数如表1所示。数据采集和分析软件为美国Thermo Fisher公司的LC quan 2.7。

**表1:** 7种PFCs的离子对、碰撞能和透镜电压

Compound	Ion pair （*m*/*z*）	Collision energy/eV	Tube lens voltage/V
Perfluorobutyric acid （PFBA）	212.9/167.0^*^	12	68
Perfluoropentanoic acid （PFPeA）	262.9/218.9^*^	11	65
Perfluorohexanoic acid （PFHxA）	312.9/268.9^*^	11	70
Perfluoroheptanoic acid （PFHpA）	362.9/318.8^*^	11	72
Perfluorooctanoic acid （PFOA）	412.9/368.9^*^	13	72
Perfluorononanoic acid （PFNA）	462.9/419.0^*^	13	75
Perfluorodecanoic acid （PFDA）	512.9/468.8^*^	13	78

* Quantitative ion.

## 2 结果与讨论

### 2.1 Fe_2_O_3_/CTF-F材料的表征

首先，本实验通过XPS对所制备的Fe_2_O_3_/CTF-F材料的化学组成进行了分析。实验结果显示，Fe_2_O_3_/CTF-F材料由碳（55.48%）、氟（17.50%）、氧（14.60%）、氮（10.16%）和铁（0.32%）组成，说明该材料具有较高的氟含量（见图1a）。此外，在Fe（2*p*）高分辨谱图中可以观察到在711.6 eV（Fe 2*p*_3/2_）和724.6 eV（Fe 2*p*_1/2_）处具有强峰，并在734.0 eV和717.8 eV处出现了卫星峰（见图1b），表明Fe_2_O_3_颗粒存在于所制备的材料中。同时，在C（1*s*）高分辨率图谱中出现了3个强峰分别对应苯环上的C=C键（284.1 eV）、三嗪环上的N=C-N键（285.5 eV）和C-F键（288.3 eV）（见图1c），证明通过四氟对苯二腈聚合形成了CTF-F材料。FT-IR分析进一步证实了CTF-F材料的形成，其中1 396.2 cm^‒1^和1 560.1 cm^‒1^处的强吸收峰是三嗪环振动的特征峰，1 068.0 cm^‒1^处的吸收峰属于C-F键的伸缩振动（见图1d）。上述实验结果证明了通过一锅法可以成功制备Fe_2_O_3_/CTF-F材料。

**图1:**
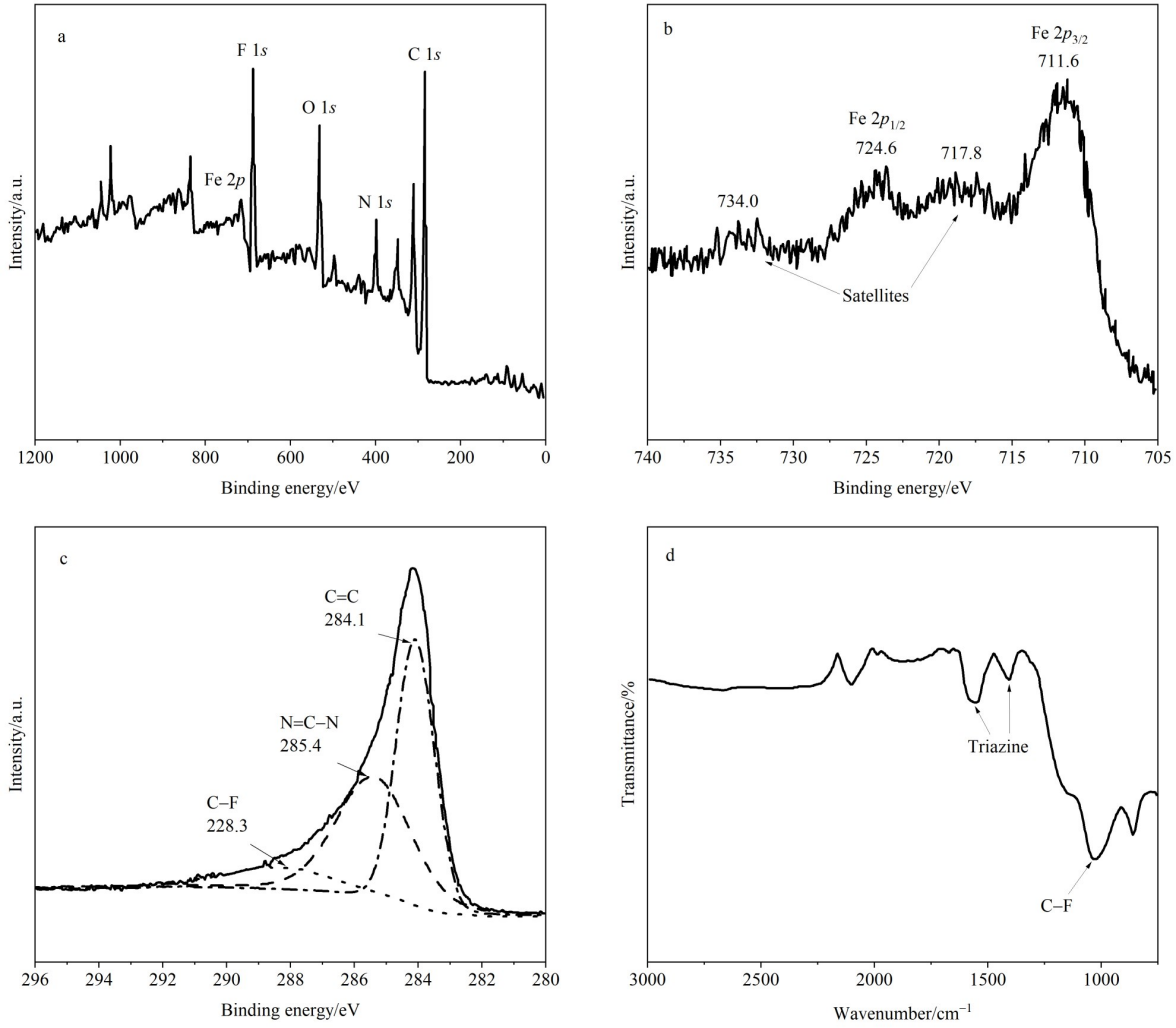
（a）Fe_2_O_3_/CTF-F的XPS图，（b）Fe（2*p*）和（c）C（1*s*）的高分辨XPS图以及（d）Fe_2_O_3_/CTF-F的FT-IR图

随后，本实验采用XRD分析了Fe_2_O_3_/CTF-F的晶体结构。如图2a所示，在26.0°处观察到一个宽峰，而在15°以下未观察到任何衍射峰，说明所形成的CTF-F以无定形结构为主。拉曼光谱中D带和G带的强度比为1.01，进一步证明CTF-F的无定形结构特征（见图2b）。此外，在XRD谱图中18.2°、30.0°、31.1°、35.3°、36.7°、43.0°和62.3°处有明显的衍射峰，其分别与*γ*-Fe_2_O₃ 的（111）、（220）、（221）、（311）、（222）、（400）和（440）晶面相匹配，表明磁性颗粒主要以*γ*-Fe_2_O₃晶形存在。本实验还通过氮气吸附-脱附实验测定了Fe_2_O_3_/CTF-F的孔隙结构。如图2c和2d所示，Fe_2_O_3_/CTF-F呈现出Ⅰ型氮气吸附-脱附等温曲线，拥有较高的比表面积（1 452.3 m^2^/g）和孔隙度（0.82 cm^3^/g）。同时，Fe_2_O_3_/CTF-F的孔径尺寸以微孔和介孔为主，可容纳分子直径小于1.38 nm的PFCs，这为PFCs的高效萃取提供了有力保障。

**图2:**
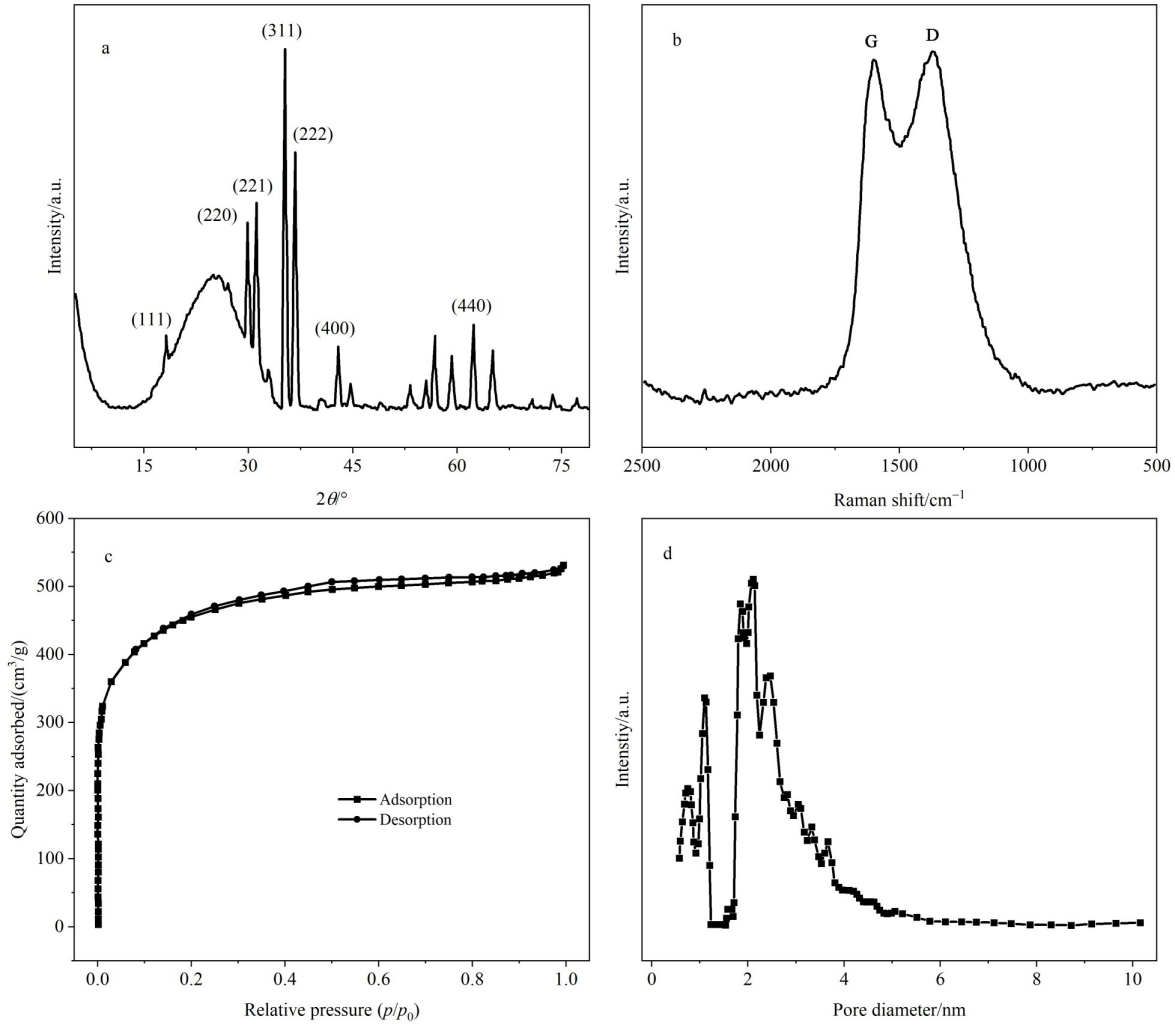
Fe_2_O_3_/CTF-F的（a）XRD图、（b）拉曼光谱图、（c）氮气吸附-脱附等温曲线以及（d）孔径分布图

最后，本实验通过VSM在室温下对Fe_2_O_3_/CTF-F的磁性能进行了表征。如图3所示，Fe_2_O_3_/CTF-F的饱和磁化强度值为7.1 emu/g，其在水中具有良好的分散性，能在90 s内实现磁性分离，能满足MSPE对磁性能的要求。

**图3:**
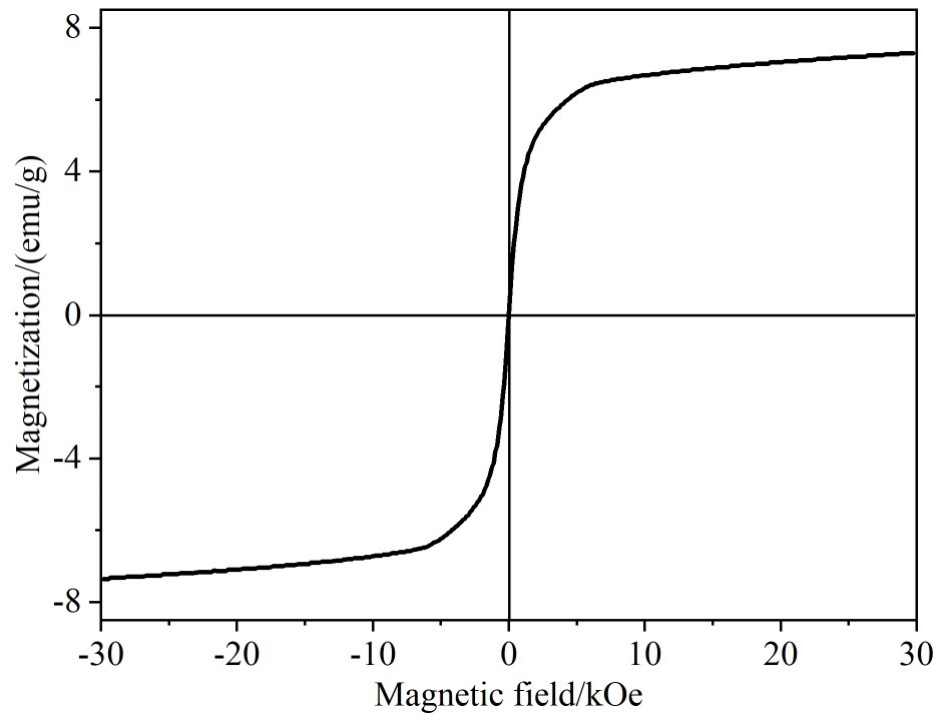
Fe_2_O_3_/CTF-F的磁滞回线图

### 2.2 MSPE过程的优化

为了获得最佳的萃取效果，通过单因素实验对MSPE过程中吸附剂用量、萃取时间、洗脱溶剂和洗脱时间进行了优化。

#### 2.2.1 吸附剂用量

本实验考察了吸附剂用量在1.0~10.0 mg范围内对萃取效果的影响。如图4a所示，随着吸附用量的增加，所有PFCs的回收率也随之增大，并且当吸附剂用量为5.0 mg时达到最大值。实验结果表明，少量的吸附剂即可对所有PFCs获得优异的萃取效果。因此，最佳的吸附剂用量为5.0 mg。

**图4:**
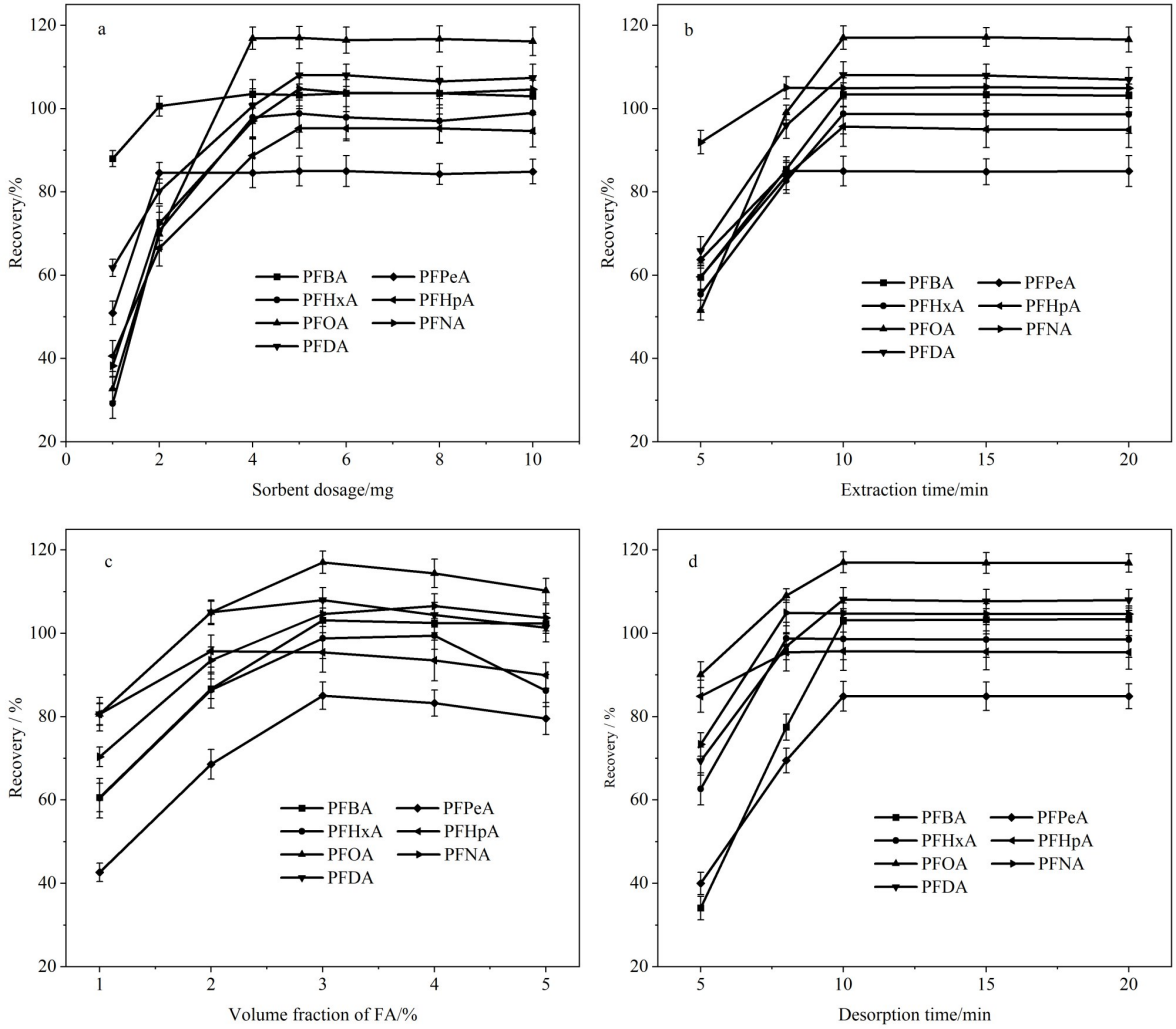
（a）吸附剂用量、（b）萃取时间、（c）洗脱溶剂中甲酸含量以及（d）洗脱时间对PFCs萃取效果的影响（*n*=3）

#### 2.2.2 萃取时间

为了保证吸附剂有充足的时间与PFCs发生相互作用，实验还考察了萃取时间在5~20 min范围内对萃取效果的影响。如图4b所示，随着萃取时间的增加，萃取回收率先是逐步上升，并在10 min时达到最大值，然后保持不变。实验结果说明，Fe_2_O_3_/CTF-F对PFCs具有出色的吸附能力，在较短时间内就能获得理想的萃取效果。因此，最佳的萃取时间为10 min。

#### 2.2.3 洗脱溶剂

理想的洗脱溶剂不仅要对PFCs有良好的溶解度，并且能够破坏PFCs与Fe_2_O_3_/CTF-F之间的相互作用。由于甲醇很难将所有PFCs从Fe_2_O_3_/CTF-F上洗脱下来，因此实验考察了含不同体积分数（1%~5%）FA的甲醇溶液对萃取效果的影响。如图4c所示，当FA体积分数达到3%时，回收率达到最大值。实验结果说明，PFCs在含3% FA的甲醇溶液中的溶解度最佳，并且FA对破坏PFCs与Fe_2_O_3_/CTF-F之间F-F相互作用起到关键作用。因此，最佳的洗脱溶剂为含3% FA的甲醇溶液。

#### 2.2.4 洗脱时间

本实验还考察了洗脱时间在5~20 min范围内对萃取效果的影响，以确保洗脱溶剂有充足的时间能够破坏PFCs与Fe_2_O_3_/CTF-F之间的作用力。如图4d所示，随着洗脱时间的增加，回收率逐渐上升，并在10 min时达到最大值；进一步增加洗脱时间，回收率无明显变化。实验结果表明，当洗脱时间不低于10 min时，洗脱溶剂能充分破坏PFCs与Fe_2_O_3_/CTF-F之间的相互作用。因此，随后的实验选定10 min作为洗脱时间。

### 2.3 方法学考察

在最优条件下，本实验将MSPE方法与HPLC-MS/MS分析仪器相结合，建立了一套分析方法用于PFCs的检测，并分别考察了该方法的线性范围、相关系数（*R*）、检出限（LOD）、定量限（LOQ）和精密度（RSD）。如表2所示，7种PFCs的线性范围为0.008~250.0 pg/mL，并且都具有良好的线性关系（*R*≥0.999 2）。实验利用最低浓度检测法测得的LOD（*S*/*N*=3）和LOQ（*S*/*N*=10）分别为0.002~0.005 pg/mL和0.008~0.020 pg/mL。此外，实验在低（0.01 pg/mL）、中（1.0 pg/mL）、高（100.0 pg/mL）3个水平下对分析方法的日内和日间精密度进行了考察。每个加标水平在日内测定5个平行样，在日间连续测定5天，测得的日内和日间RSD（*n*=5）分别为3.4%~7.6%和2.8%~8.2%，说明所建立的分析方法具有良好的稳定性。

**表2:** 所建立方法的线性范围、相关系数、检出限以及精密度

Compound	Linear range/ （pg/mL）	*R*	LOD/（pg/mL）	RSDs/% （*n*=5）	
				Intra-day	Inter-day
PFBA	0.010-250.0	0.9992	0.004	6.1， 5.1， 8.2	6.6， 6.8， 7.6
PFPeA	0.020-250.0	0.9996	0.005	3.8， 7.3， 6.2	5.5， 5.4， 6.8
PFHxA	0.010-250.0	0.9996	0.004	5.2， 7.4， 4.8	3.4， 5.4， 7.3
PFHpA	0.020-250.0	0.9992	0.005	4.5， 5.6， 6.8	6.6， 5.8， 6.9
PFOA	0.008-250.0	0.9996	0.002	6.8， 4.2， 6.2	5.5， 6.4， 6.8
PFNA	0.010-250.0	0.9993	0.004	5.8， 4.6， 6.7	4.2， 5.4， 6.6
PFDA	0.008-250.0	0.9998	0.002	4.7， 2.8， 5.2	4.4， 6.4， 7.0

Intra-day and inter-day RSDs were obtained from left to right at spiked levels of 0.01， 1.0 and 100.0 pg/mL， respectively.

### 2.4 抗基质干扰试验

软饮料样品基质较为复杂，其通常含有一些干扰物，如蛋白质、糖类、氨基酸、盐类等，这可能会对萃取过程产生不利影响。因此，本实验考察了蔗糖、葡萄糖、柠檬酸、维生素C、L-半胱氨酸、牛血清蛋白、阳离子（Na^+^， K^+^和Ca^2+^）和阴离子（Cl^‒^， CO_3_^2‒^和H_2_PO_4_^‒^）在不同浓度下对方法回收率的影响（见表3）。评估这些物质是否具有干扰作用以方法回收率（250.0 pg/mL PFCs）的相对误差不超过10%作为标准，耐受限度即为无显著影响下的干扰物最大允许浓度。实验结果说明，Fe_2_O_3_/CTF-F与PFCs之间产生的F-F相互作用具有优异的选择性，能够很好地抵抗基质中存在的多数干扰物。

**表3:** PFCs萃取的耐受限度

Interferents	Tolerance limit/（mmol/L）
Sucrose	100.0
Glucose	50.0
Na^+^， K^+^， Cl^‒^， Ca^2+^	20.0
CO_3_^2‒^， H_2_PO_4_^‒^	10.0
Citric acid	5.0
Vitamin C， L-cysteine	1.0
Bovine serum albumin	0.5

### 2.5 与其他方法的比较

为了进一步综合评价所建立分析方法的优越性，将其与已报道的方法进行了全面的比较。如表4所示，本方法仅需少量的吸附剂（5.0 mg），经过较短时间的预处理（20 min）就能获得较低的检出限（0.002 pg/mL）。这可能是因为Fe_2_O_3_/CTF-F提供了大量可接触吸附位点，与PFCs之间发生了强F-F相互作用，并拥有快速磁性分离的能力。比较结果说明，所建立的MSPE-HPLC-MS/MS方法具有快速、灵敏、准确的优点，能够用于软饮料中PFCs污染的监测。此外，本研究中吸附剂的制备方法非常简单、便捷，有利于该方法的转化应用。

**表4:** 本方法与其他已报道HPLC-MS/MS方法的比较

MSPE adsorbent	Adsorbent dosage/mg	Pretreatment time/min	LOD/（pg/mL）	Ref.
Fe_3_O_4_-PLS Fe_3_O_4_@EB-iCOFs	50.0	16^*^	0.001-0.620	［^11^］
M-Ny6	10.0	65	1.200	［^12^］
Fe_3_O_4_@C	44.0	46^*^	0.030-0.090	［^13^］
M-FPCs	8.0	35	0.020-0.160	［^14^］
Fe_3_O_4_@EB-iCOFs	20.0	15^*^	0.100-0.800	［^15^］
MFCA	30.0	27^*^	0.010-0.036	［^16^］
Fe_3_O_4_@TpPa-F_4_	20.0	30^*^	0.005-0.050	［^4^］
CTF/Fe_2_O_3_ （CTF）/Fe_2_O_3_	50.0	21^*^	0.620-1.390	［^17^］
Fe_2_O_3_/CTF-F	5.0	20	0.002-0.005	this work

* Need to be dried under nitrogen gas. Fe_3_O_4_-PLS： magnetic polystyrene pyrrolidone； M-Ny6： magnetic nylon 6 nanocomposite； Fe_3_O_4_@C： magnetic carbon nanotube composite nanospheres； M-FPCs： magnetic and fluorinated porous carbons； Fe_3_O_4_@EB-iCOFs： magnetic ionic covalent organic framework； MFCA： magnetic fluorinated carbon nanotubes adsorbent； Fe_3_O_4_@TpPa-F_4_： fluorinated magnetic covalent organic frameworks； CTF/Fe_2_O_3_： magnetic covalent triazine-based framework composites.

### 2.6 实际样品分析

为了评价所建立MSPE-HPLC-MS/MS方法对实际样品中7种PFCs的检测能力，实验分析了5种软饮料，包括矿泉水、果汁、汽水、能量饮料和茶饮料。图5为茶饮料样品的色谱图。

**图5:**
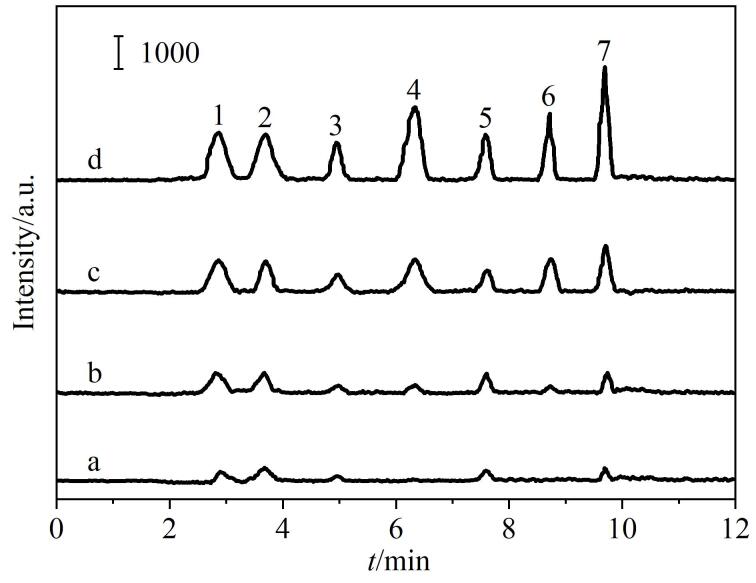
茶饮料样品及其加标样品的色谱图

如表5所示，所有样品均检测出了PFCs，含量范围为3.5~54.6 pg/mL。其中，测得的全氟辛酸（PFOA）最高含量为54.6 pg/mL，其未超出国家标准GB 5749-2022的限量（80 pg/mL）。同时，为进一步考察该方法的精密度和准确度，分别在低（1.0 pg/mL）、中（20.0 pg/mL）、高（100.0 pg/mL）3个水平下进行了实际样品加标试验，得到的加标回收率为81.9%~110.5%，RSD均低于10%（*n*=5）。实验结果说明，所建立的分析方法具有良好精密度和准确度，适合用于软饮料中7种PFCs的高灵敏检测。

**表5:** 7种PFCs的加标回收率及精密度（*n*=5）

Sample	Compound	Found/（pg/mL）	Recoveries （RSDs）/%		
			1.0 pg/mL	20.0 pg/mL	100.0 pg/mL
Mineral Water	PFBA	N.D.	85.8 （2.3）	97.1 （5.2）	93.6 （3.7）
	PFPeA	22.7	100.6 （5.3）	96.5 （4.3）	81.9 （1.8）
	PFHxA	N.D.	96.5 （5.1）	98.6 （7.1）	105.6 （4.1）
	PFHpA	N.D.	82.6 （7.2）	98.2 （5.1）	94.6 （4.2）
	PFOA	54.6	96.5 （4.8）	106.9 （6.2）	93.1 （9.8）
	PFNA	20.5	89.6 （5.2）	99.5 （6.3）	96.9 （8.5）
	PFDA	16.7	101.5 （5.1）	95.6 （4.5）	86.6 （4.9）
Juice	PFBA	N.D.	101.2 （4.5）	89.4 （6.5）	95.5 （2.5）
	PFPeA	N.D.	92.3 （3.9）	96.3 （1.8）	97.8 （5.2）
	PFHxA	48.1	91.4 （2.6）	105.7 （9.8）	101.6 （7.1）
	PFHpA	3.5	83.6 （8.1）	99.8 （7.2）	87.6 （5.1）
	PFOA	27.5	86.2 （8.2）	105.9 （7.3）	101.6 （4.2）
	PFNA	14.5	103.5 （8.4）	108.6 （7.1）	100.6 （4.8）
	PFDA	3.6	108.9 （7.5）	94.7 （4.9）	93.6 （6.3）
Sodas	PFBA	N.D.	110.5 （2.5）	98.5 （6.4）	96.4 （3.1）
	PFPeA	8.9	87.5 （5.8）	91.6 （6.1）	94.3 （8.1）
	PFHxA	15.2	101.3 （6.5）	99.5 （4.2）	102.3 （3.3）
	PFHpA	15.6	96.1 （2.1）	91.6 （3.8）	97.1 （7.2）
	PFOA	26.8	99.4 （7.5）	87.1 （8.6）	86.9 （4.1）
	PFNA	23.4	101.6 （6.2）	106.3 （6.3）	98.1 （7.1）
	PFDA	13.8	89.6 （7.1）	101.7 （8.5）	102.5 （6.2）
Energy drinking	PFBA	31.8	86.5 （7.5）	96.6 （3.5）	83.1 （2.3）
	PFPeA	5.6	98.3 （3.1）	97.1 （4.2）	105.2 （5.1）
	PFHxA	16.3	87.4 （4.7）	95.3 （5.2）	97.3 （7.2）
	PFHpA	20.5	101.3 （7.4）	96.5 （6.7）	86.3 （5.2）
	PFOA	9.5	105.2 （7.3）	103.7 （8.2）	100.3 （6.7）
	PFNA	42.6	108.1 （6.4）	104.2 （5.7）	96.8 （1.2）
	PFDA	43.4	104.2 （3.2）	95.3 （1.5）	98.4 （3.9）
Tea drinking	PFBA	45.8	99.6 （5.1）	98.5 （6.3）	105.9 （4.6）
	PFPeA	8.5	84.8 （4.3）	96.3 （2.8）	98.1 （5.1）
	PFHxA	21.5	101.5 （6.3）	103.5 （7.2）	99.6 （5.5）
	PFHpA	N.D.	97.1 （7.2）	98.5 （4.1）	83.6 （4.3）
	PFOA	18.8	89.3 （3.1）	86.3 （2.3）	98.1 （5.4）
	PFNA	N.D.	101.6 （4.6）	94.5 （3.9）	93.8 （5.1）
	PFDA	43.7	98.5 （8.5）	91.1 （4.3）	87.4 （4.9）

N.D.： not detected.

## 3 结论

在本实验中，通过一锅法成功制备了一种磁性氟化共价三嗪骨架材料（Fe_2_O_3_/CTF-F），并将其作为MSPE吸附剂，用于7种PFCs的高效萃取。Fe_2_O_3_/CTF-F拥有高比表面积、高孔隙度和高含氟量，继而对PFCs表现出优异的萃取能力。随后，将MSPE方法与HPLC-MS/MS技术相结合，建立了一种简单、快速、灵敏的分析方法，并成功地应用于实际软饮料样品中7种PFCs的检测。综上所述，所建立的分析方法在对PFCs分析检测方面具有良好的应用前景。
